# Effect of Isosteviol on Wheat Seed Germination and Seedling Growth under Cadmium Stress

**DOI:** 10.3390/plants10091779

**Published:** 2021-08-26

**Authors:** Liang Zhang, Bingbing Gao

**Affiliations:** 1 School of Chemistry and Bioengineering, Taizhou College, Nanjing Normal University, Taizhou 225300, China; 2 School of Pharmaceutical Sciences, Nanjing Tech University, Nanjing 211816, China

**Keywords:** cadmium stress, wheat, isosteviol, physiological property, alleviation

## Abstract

Cadmium (Cd) contamination of agricultural soil has become a serious threat to global food security. The present study highlights the effect of added isosteviol in modulating growth physiology and antioxidant defense systems conferring tolerance against cadmium (Cd) stress in wheat. Wheat growth, chlorophyll content, malondialdehyde (MDA) content of leaves, dehydrogenase activity of root, and antioxidant enzyme activity were determined to get an overview of cellular response in conquering Cd-induced oxidative stress damages. The results indicated that wheat germination was inhibited under Cd^2+^ concentration at 10 µM. The presence of isosteviol and gibberellic acid (GA) significantly alleviated the inhibitory effect on the growth of wheat seedling under 10 µM Cd^2+^ stress. Moreover, different concentrations of isosteviol and GA regulated the physiological changes of wheat under Cd stress: more chlorophyll *a* + *b* content; less MDA content; and higher dehydrogenase activity of root and antioxidant enzyme activity of superoxide dismutase (SOD), peroxidase (POD), and catalase (CAT), as compared to Cd alone in wheat seedling. The present study thus suggests a possible role of isosteviol in amelioration of Cd stress by increasing chlorophyll content and root dehydrogenase activity, which also could reduce oxidative damage of the cell membrane by regulating the activities of antioxidant enzymes in wheat seedling.

## 1. Introduction

Cadmium (Cd), a non-essential element in plant’s metabolic processes, is recognized as a harmful heavy metal that can be found in contaminated soils. Cd is unnecessary in the growth of plants; plant roots actively take up Cd ions, which are then transported to vegetative parts located above the ground [1,2,3]. Cd has been observed to seriously inhibit crop production and product quality beyond a certain level [4]. Hence, Cd is detrimental for plant cells and creates fluctuations in plant’s growth, causing the distortion of membranes, generation of reactive oxygen species (ROS), problems in respiration, as well as inhibition of photosynthesis [5,6]. Therefore, developing feasible technology to reduce Cd stress is quite important. One method selects and cultivates species as well as genotypes that have high tolerance to Cd, and this has been stated in cases of rice, durum wheat, maize, and oat cultivation [2,3,7,8]. Moreover, calcium, a necessary element in crop growth, has been adopted in alleviating the toxicity of Cd [9,10]. Exogenous calcium can be applied to ameliorate the injury caused by Cd stress by reducing Cd absorption in plants.

**Figure 1:**
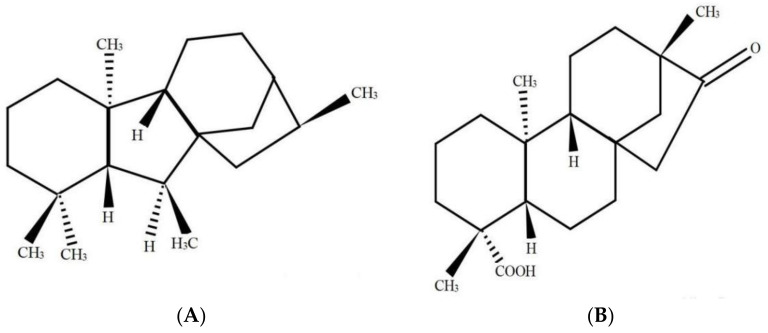
Structural formulas of gibberellins (**A**) and isosteviol (**B**).

Some plant growth regulators (PGRs), such as abscisic acid (ABA), indole-3-acetic acid (IAA), gibberellins (GAs), as well as salicylic acid (SA), have been recognized to exert crucial functions to activate the defensive system in plants under a stressful environment [11,12,13]. Notably, the crosstalking in the external as well as internal signaling in the transduction pathways within plant cells has made it possible to use technologies aiming at increasing the resistance of plants towards abiotic as well as biotic stresses posed by the environment [14]. At present, in the research field of PGRs, GAs are applied widely. Studies have shown that exogenous application of gibberellic acid (GA) could effectively alleviate Cd stress effects in wheat through Cd immobilization by increasing the contents of free proline and soluble phenol detoxification of Cd within the plant cells [12]. According to Liang et al. (2021), GA sprayed on leaves of *Sedum alfredii* Hance increased plant growth. In addition, the presence of GA enhanced chlorophyll *a*, carotenoid, and potassium contents in leaves and decreased the malondialdehyde (MDA) content [15]. GAs is a diterpene acid derived from tetracyclic skeleton. Isosteviol, which is referred to as the ent-16-ketobeyeran-19-oic acid, has been a kind of beyerane diterpene that can be extracted from stevioside through acid hydrolysis (Figure 1). Stevioside has been used as a conventional non-caloric sweetener, which can be isolated from *Stevia rebaudiana* [16]. Both GAs and isosteviol have been derived through secondary metabolism, creating intermediates of mevalonic acid, kaurenoic acid etc. Also, they exhibit striking similarities in their parent structure [17].

In previous research, there has been noteworthy evidence of steviol glycosides derivatives showing gibberellin-like activity, which could regulate the activities of antioxidant enzymes and photosynthetic system of plants in adverse environments [18,19,20], and then scavenge the excess levels of ROS, produced by oxidative insults [21,22]. In heavy metal-polluted agricultural lands, it is very necessary to reduce the toxicity and accumulation of pollutants through the development and application of new PGRs. However, no meticulous investigation has so far been carried out to untie the underlying physiological activity of isosteviol-mediated Cd stress alleviation in wheat, the world’s second most important cereal crop after rice. Hence, there is an urgent need to evaluate the physiological responses of wheat seedling towards Cd stress in the presence of added exogenous isosteviol under hydroponic conditions. Our experiments involved wheat (*Triticum aestivum* L. cv. Yannong 19) as a testing crop, which was a wheat variety with low accumulation ability of Cd in grains. We preliminarily studied the effect of isosteviol in wheat germination as well as the seedling growth in the presence of Cd stress. This was done in order to scientifically research the effect of isosteviol in alleviating the heavy metal-induced toxicity on the growth of crops under stress. In addition to studying growth indexes of wheat, chlorophyll content, MDA content, root dehydrogenase, and antioxidant enzyme activity under Cd stress in the presence or absence of added isosteviol, were studied in detail to get an overview of cellular response in conquering Cd-induced oxidative stress damages. Overall, this investigation provides a deeper understanding of the physiological response mechanism of Cd stress alleviation by added isosteviol in wheat.

## 2. Results

### 2.1. Cd Stress Effect on Germination as Well as the Growth of Seedlings

Table 1 shows the final germination (FG) rate, germination index (GI), vigor index (VI), shoot length (SL), root length (RL), and fibrous root number (FRN) measured on day 7 after sowing. It is evident that values for FG rate, VI, SL, and FRN were reduced as the Cd^2+^ concentration was elevated. At the 1 µM concentration of Cd^2+^, compared to the control, only VI decreased significantly, and other growth indexes had no significant changes. Meanwhile, with the increase of Cd^2+^ concentration, the overall trend of all indicators decreased.

The treatment of 10 µM Cd^2+^ concentration showed significant inhibitory effect, which led to the lower FG rate, GI, SL, RL, and FRN. Therefore, as compared to control, the results of the treatment group were reduced by 5.2%, 20.5%, 9.1%, 6.3%, and 5.8%, respectively. However, as compared to the control, the treatment of 1 µM Cd^2+^ concentration only reduced VI by 2.3%, and there was no significant difference in other indexes.

Based on those above-stated results, germination of wheat seeds was significantly inhibited when the concentration of Cd^2+^ was 10 µM. Therefore, wheat seeds were treated with 10 µM Cd^2+^ concentration with an aim to examine the effect of isosteviol in wheat seed germination under Cd stress.

### 2.2. Effects of Isosteviol on Seed Germination and Seedling Growth of Wheat under Cd Stress

Table 2 shows the values obtained for FG rate, GI, VI, SL, RL, and FRN on the 7th day post sowing; isosteviol had a significant impact on seed germination and seedling growth of wheat under Cd stress. Compared to Cd alone, the treatment of isosteviol and GA improved the germination rate and seedling growth index of wheat in varying degrees, but they were significantly lower than those in the control.

In all treatments of isosteviol, 10^−8^ M isosteviol concentration significantly increased FG rate, VI, SL, RL, and FMN. As compared to Cd alone, it increased by 2.8%, 19.0%, 10.2%, 13.4%, and 9.3% respectively. In addition, the concentration of 10^−9^ M isosteviol significantly increased GI, as compared to Cd alone, it increased by 6.5%. The results showed that isosteviol treatment could alleviate the inhibition of wheat seed germination and early seedling growth under Cd stress. Moreover, the effects of isosteviol and GA on wheat germination were different under Cd stress.

### 2.3. Effects of Isosteviol on Chlorophyll Content in Wheat Leaves under Cd Stress

Data for chlorophyll *a*, chlorophyll *b*, chlorophyll *a* + *b* contents, and chlorophyll *a*/*b* ratio of wheat seedlings are shown in Table 3. Leaves of Cd-challenged wheat showed drastic reduction in chlorophyll *a* (~1.9-fold as compared to control leaf), chlorophyll *b* (~1.8-fold), and chlorophyll *a* + *b* (~1.8-fold) contents. However, treatments of isosteviol and GA could slow down this decrease under Cd stress. Particularly, the 10^−8^ M concentration of isosteviol and 10^−5^ M concentration of GA significantly increased chlorophyll *a* + *b* content, as compared to Cd alone, which were increased by 30.8 and 23.1%, respectively. And there was no significant difference between them. In addition, compared to the Cd alone, the chlorophyll *a* content was significantly increased with 10^−8^ M concentration of isosteviol treatment under Cd stress. However, all treatments did not show any significant deviation in chlorophyll *a*/*b* ratio from the control.

### 2.4. Effects of Isosteviol on MDA Content in Wheat Leaves under Cd Stress

**Figure 2:**
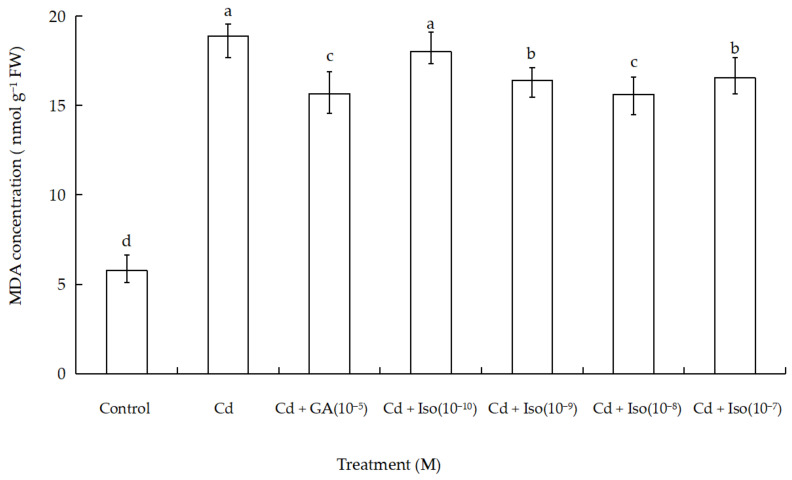
Effects of isosteviol on MDA content in wheat (*Triticum aestivum* L. cv. Yannong 19) leaves under Cd stress. MDA: malondialdehyde; Cd: cadmium; GA: gibberellin acid; Iso: isosteviol; M: isosteviol and gibberellic acid concentration unit (mol L^−1^); FW: fresh weight. Data are presented in the form of mean ± S.D. (*n* = 12 in each group); different letters are indicative of the presence of significant difference (*p <* 0.05) between different treatments.

MDA content was used as a general marker of stress-induced oxidation damage to lipid membranes. The treatment of Cd alone (10 µM) caused significant increase in MDA content of wheat leaves (Figure 2). As shown in Table A1 in the Appendix A, maximum increase (~3.3-fold as compared to control) was determined in leaves of the treatment of Cd alone. However, comparatively less membrane damage (~2.7-fold) was noticed in isosteviol (10^−8^ M) supplemented with Cd-exposed wheat.

### 2.5. Effects of Isosteviol on Dehydrogenase Activity of Root in Wheat Seedlings under Cd Stress

**Figure 3:**
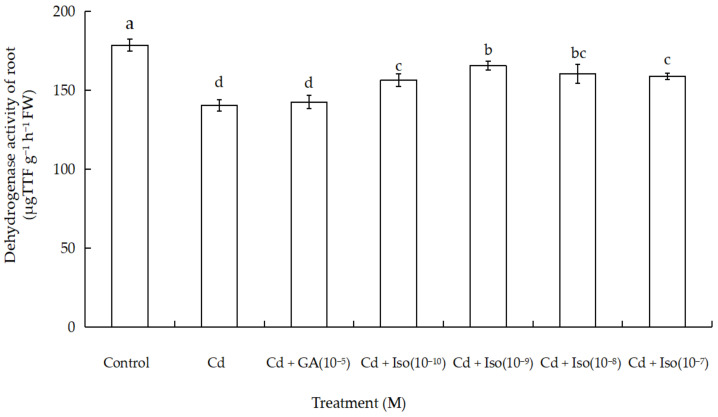
Effects of isosteviol on dehydrogenase activity of wheat (*Triticum aestivum* L. cv. Yannong 19) root under Cd stress. Cd: cadmium; GA: gibberellin acid; Iso: isosteviol; TTF: triphenyltetrazolium formazan; M: isosteviol and gibberellic acid concentration unit (mol L^−1^); FW: fresh weight. Data are presented in the form of mean ± S.D. (*n* = 12 in each group); different letters are indicative of the presence of significant differences (*p <* 0.05) between different treatments.

Data for dehydrogenase activity of root in wheat seedlings are shown in Figure 3. Cd exposure caused significant decrease in dehydrogenase activity of wheat seedlings root. Maximum reduction (~21.4% as compared to control) was noticed in the root of the treatment of Cd alone. However, comparatively more dehydrogenase activity of root (7.3%) was noticed in isosteviol (10^−9^ M) supplemented with Cd-exposed wheat.

### 2.6. Effects of Isosteviol on Antioxidant Enzyme Activity of Wheat Seedlings under Cd Stress

**Figure 4:**
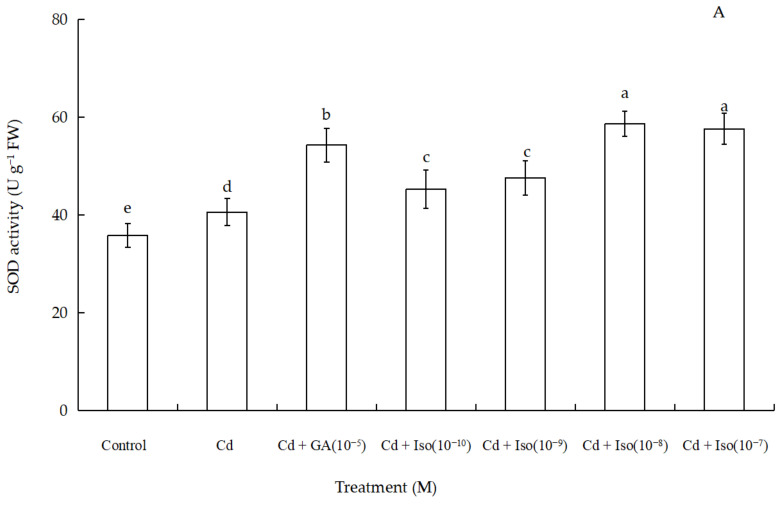

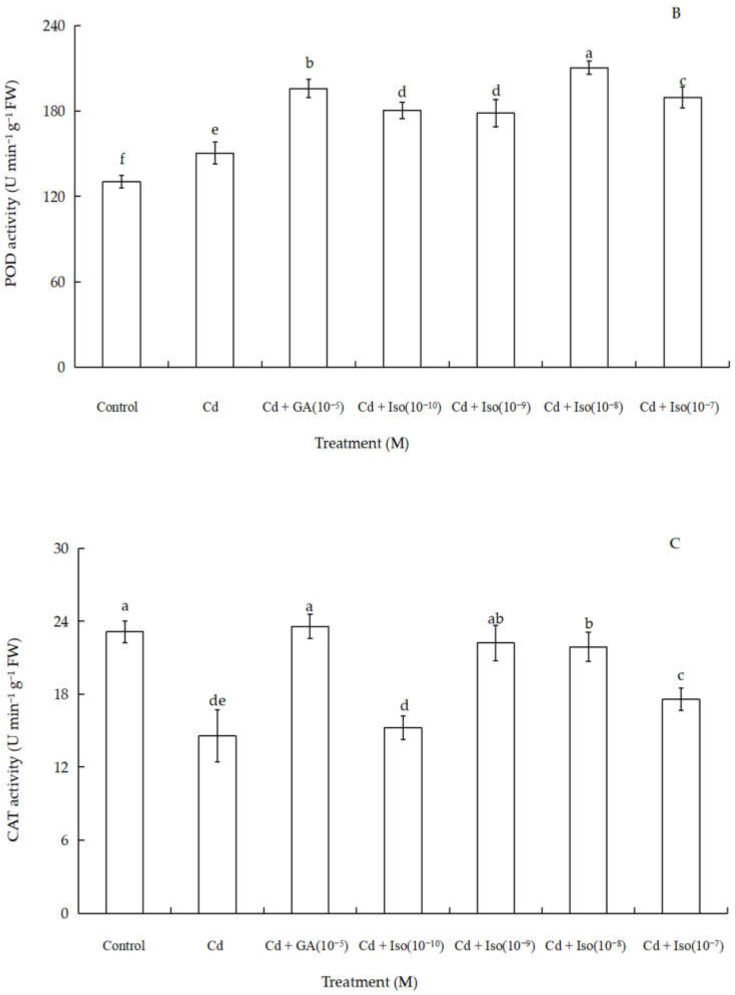
Effects of isosteviol on the activity of SOD (**A**), POD (**B**), and CAT (**C**) of wheat (*Triticum aestivum* L. cv. Yannong 19) seedlings under Cd stress. SOD: superoxide dismutase; POD: peroxidase; CAT: catalase; Cd: cadmium; GA: gibberellin acid; Iso: isosteviol; M: isosteviol and gibberellic acid concentration unit (mol L^−1^); FW: fresh weight. Data are presented in the form of mean ± S.D. (*n* = 12 in each group); different letters are indicative of the presence of significant differences (*p <* 0.05) between different treatments.

Data for the activity of superoxide dismutase (SOD), peroxidase (POD), and catalase (CAT) of wheat seedlings are shown in Figure 4. Cd stress had a significant impact on foliar antioxidant enzymes activities. Out of the three studied antioxidant enzymes, SOD and POD activity were significantly increased (~63.7% and 61.1% as compared to control leaf, respectively) in the treatment of 10^−8^ M concentration isosteviol (Figure 4A,B). In contrast, the treatment of Cd alone leaves showed marked decline (~36.8%) in CAT activity (Figure 4C). Nevertheless, compared to the control, 10^−9^ M concentration of isosteviol and 10^−5^ M concentration of GA had no significant difference in leaf CAT activity. The results showed that the treatment of isosteviol could regulate the activity of antioxidant enzymes in the cotyledons of wheat seedlings under Cd stress, which could reduce the adverse effects caused by the accumulation of ROS.

## 3. Discussion

The adaptation of plants to heavy metal stress is a highly complex physiological response. In the process of evolution, plants have developed efficient homeostatic mechanisms to regulate the absorption, accumulation, transport, and detoxification of metals. Therefore, it is still the focus of attention. Changes in germination of seeds as well as the growth of seedlings are the main observable plant processes that can reflect the influence of heavy metal toxicity. Generally, it is considered that heavy metal can play a stimulating effect under a low concentration, but it can exert an inhibiting effect under a high concentration in regard to its effect in seed germination as well as the growth of seedling [23,24,25]. Also, it has been reported that Cd promotes growth of seedlings in rice (*Oryza sativa* L.) within the range of 0.05‒0.1 µM. Additionally, Cd exhibited an inhibiting action for seedling root and hypocotyl growth at 0.5‒1 µM [26]. It is mainly because most of the Cd taken up by plant cells is accumulated in the roots and only a small amount is transported to shoots. Once Cd enters the cell, it alters normal cellular functions by replacing Ca^2+^, Zn^2+^, and Fe^2+^ from proteins, as these elements display chemical similarity to Cd [27]. According to Kuriakose and Prasad (2008), sorghum could tolerate Cd^2+^ at a concentration of as high as 0.5 mM. At the Cd concentration of >3.0 mM, the germination of seeds was impacted negatively, and the growth of seedlings completely ceased. Meanwhile, the data obtained for Cd effect on plant growth suggest that the activities of hydrolases were markedly decreased with the increase of Cd^2+^ concentration [28]. It was found in this study that all wheat growth indexes were not markedly suppressed, except for VI, when Cd^2+^ concentration was at 10^−6^ M. However, when the Cd^2+^ concentration was higher than 10 µM, the growth indexes (such as FG rate, GI, SL, RL, and FRN) of wheat were significantly inhibited. With an increase in Cd^2+^ concentration, the inhibition effect became more apparent. Therefore, in order to explore the effect of isosteviol on wheat seed germination under Cd stress, we selected 10 µM Cd^2+^ concentration for follow-up experiments.

Many studies have indicated GA’s active effect in regard to alleviating heavy metal stress by improving wheat germination rate and growth of seedlings [29,30]. Moreover, isosteviol shows similarities with GAs in their matrix structure [17]. According to Liu et al. (1994), it showed that isosteviol and steviol could promote the germination of rice seeds, and their activities were better than GA [31]. Our findings revealed that the treatment of Cd exposure and isosteviol (Cd + Iso) could have positive effects in promoting the growth indexes of wheat seeds, compared to Cd exposure alone. Notably, when wheat seeds were treated with 10^−8^ M concentration of isosteviol under 10 µM Cd^2+^ stress, similar results were obtained with GA treatment for FG rate. Studies showed that the exogenously applied GA could mitigate Cd stress, as observed within *Glycine Max*, which was particularly true at the Cd^2+^ treatment concentration of 5 µg mL^−1^ in the plants [32]. Exogenous GA treatment could increase the fresh weight of plants under Cd stress [33]. In addition, the 10^−8^ M concentration of isosteviol significantly increased some indexes of wheat seedlings as compared to Cd alone, including VI, SL, RL, and FMN. Some studies have shown that stevioside (10^–8^ M) reduces the effect of Cd^2+^ and Zn^2+^ on plant growth and changes the activity of lectins, thereby leading to an increase in leaf length by 14% and root length by 18%, in comparison with the findings obtained for the control group, indicating that it has a protective effect on winter wheat under the stress caused by heavy metals [19,20,34]. This clearly illustrates isosteviol’s effect in alleviating the toxicity of Cd to plant roots. In this research, isosteviol, a kind of beyerane diterpene, was extracted from stevioside through acid hydrolysis, so that it would have a similar mechanism in alleviating heavy metal stress as stevioside, which can promote germination rate and seedling growth of wheat under heavy metal stress.

Chlorophyll is essential for capturing light energy in plants, thus playing a central role in plant photosynthesis. Its content directly influences energy collection for plant photosynthetics, thus affecting the accumulation of net photosynthetic energy. Ultimately, it influences plant yield and biomass [35,36]. Cd^2+^ concentration was found in previous research to be negatively correlated with the concentration of plant leaf photosynthetic pigment content [37]. It was suggested that Cd stress was detrimental to the synthesis of plant photosynthetic pigment, which would inevitably reduce the photosynthetic efficiency of plants [38]. On the other hand, according to some research, PGRs application could promote chlorophyll content generation in leaves of rape plant under Cd stress [39]. In addition, PGRs could markedly affect chlorophyll content in plants in the presence of medium Cd stress (50 μM Cd^2+^), wherein the JA and ABA significantly increase chlorophyll content by 61.4% as well as 67.1%, respectively [33,40]. Our experiment suggested that, compared to Cd alone, the treatment of Cd exposure and isosteviol (Cd + Iso) could increase chlorophyll *a* and chlorophyll *a* *+* *b* contents in leaves of wheat seedling. Particularly, 10^−8^ M concentration of isosteviol had the most significant effect on increasing chlorophyll *a* and chlorophyll *a* *+* *b* contents under Cd stress. This exhibits promotion in the synthesis of chlorophyll within seedlings leaves under 10 µM Cd^2+^. Thus, appropriate concentrations of GA and isosteviol are observed to be beneficial for enhancing the accumulation of carbohydrates in leaves, which is necessary for reducing the stress caused by heavy metal, such as Cd.

Under the conditions of biotic or abiotic stress for plant organs, free radical toxicity was observed to cause plant cell membrane lipid peroxidation (LPO) [41]. MDA, a product of LPO, can cross link with proteins, nucleic acids, amino acids, and other substances to form insoluble compounds, thus interfering with the normal life activities of cells [42,43]. In addition, MDA content is one of the important indexes to gauge membrane system damage, thus allowing reflection on the conditions of free radical activity in the body to a certain extent [44,45]. Some research articles reported that external SA decreases MDA concentration and antioxidant enzyme activity under Cd stress, thus improving the tolerance to Cd among plants [46]. Our experimental investigation demonstrated that Cd alone caused marked increase in MDA concentration, which is indicative of high membrane injury. Nevertheless, an appropriate concentration of isosteviol could reduce damage of wheat seedling cell membrane under Cd stress. Particularly, the effect of 10^−8^ M concentration of isosteviol was the most significant. Therefore, wheat seeds treated with isosteviol under 10 µM Cd^2+^ stress demonstrated reduction in MDA content, hence helping to reduce plant membrane LPO. The treatment of isosteviol could alleviate the adverse effects of Cd stress on the growth of wheat seedlings and effectively protect the plant cell membrane system.

Dehydrogenase activity of root can serve as a physiological indicator, which reflects the plant ability for water and nutrient absorption, and participate in the redox reaction of plant roots [47]. According to research, when Cd^2+^ concentration reaches 1 mg L^−1^, dehydrogenase activity of root is significantly reduced in rice. Therefore, the appropriate concentration of exogenous calcium (0–320 mg L^−1^) significantly alleviates the damage to rice roots under Cd stress [48]. As suggested by our findings, the treatment of Cd exposure and isosteviol (Cd + Iso) could increase dehydrogenase activity of root under Cd stress. Particularly, the effect of 10^−9^ M concentration of isosteviol was the most significant, which was necessarily beneficial for the survival of wheat seedling under Cd stress. Accordingly, under Cd stress, dehydrogenase activity of root was inhibited to a certain degree. Dehydrogenase activity of root reflects the sustenance of life for a plant and is also related to a plant’s capacity for stress tolerance. After isosteviol soaking treatment, the toxicity of heavy metal, Cd, to dehydrogenase activity of root in wheat seedling, exhibited a certain relieving effect.

Under the normal physiological action, the production and removal of ROS maintain a dynamic balance in plants. However, under biotic or abiotic stress conditions, plants accelerate the production of ROS, and excess ROS causes membrane LPO to plant cells [49]. Due to the excessive accumulation of ROS, the antioxidant enzyme system will be activated in plants, which can effectively remove the excess ROS such as H_2_O_2_, hydroxyl radicals, and superoxide anions etc. in plants [34,50,51]. Antioxidants have been considered as the first line of defense to mitigate heavy metal-induced oxidative stress injuries in plants. Unlike other toxic heavy metals, Cd is unable to participate in the Fenton-type reactions. However, a lot of evidence indicate that this non-redox metal indirectly triggers ROS generation by inactivation or down regulation of the ROS scavenging antioxidant enzymes or disrupting the electron transport chain [52,53,54]. It was reported that under Cd and Zn stresses, stevioside could increase the activity of antioxidant enzymes in winter wheat leaves. In addition, GA could increase the antioxidant enzyme activity of SOD, POD and CAT and reduce superoxide anion content in rice leaves under salt stress [55,56]. As suggested by our findings, Cd stress had a significant impact on foliar antioxidant enzymes activities. Compared to the control group and Cd alone, treatments of isosteviol and GA (10^−5^ M) significantly increased the activities of SOD and POD. Moreover, SOD and POD regulated the intracellular levels of hydrogen peroxide (H_2_O_2_) and superoxide anion. This showed that isosteviol had a positive effect on scavenging ROS in cells. Nevertheless, compared to the control, both 10^−9^ M concentration of isosteviol and 10^−5^ M concentration of GA had no significant difference in CAT activity. Accordingly, this indicated that isosteviol could promote the synthesis of antioxidant enzymes in wheat seedlings under Cd stress. The higher antioxidant enzyme activity was beneficial to the removal of reactive oxygen species, and this was consistent with many previous reports [49,57,58].

Cd is a nonessential metal element for plants, which has a similar cation transport system, such as members of ZIP/IRT-like proteins or Ca^2+^ channels and transporters, which are normally involved in the uptake of essential elements, such as Zn^2+^, Mg^2+^, Fe^2+^, and Ca^2+^ [59,60]. The transcriptional abundance of ZIP4 gene, mainly encoding zinc transporter in maize, was found to be significantly higher [51]. In addition, studies have shown that ZIP4 was up-regulated in root and shoot transcription in zinc-limited *Arabidopsis* plants [61]. In addition, some studies showed that with the increase of Cd^2+^ treatment concentration, the Cd^2+^ content and accumulation in root and the above ground part of wheat seedlings increased, but the ratio of Cd^2+^ content between leaves and root decreased, indicating that the root had a strong ability to accumulate Cd^2+^ [62]. However, the addition of PGRs could significantly reduce the accumulation of Cd^2+^ in winter wheat grains and leaves [20]. Hence, further investigations need to be undertaken to resolve the underlying molecular mechanism of Cd uptake in wheat, so as to clarify the role of isosteviol in it.

## 4. Conclusions

In summary, our experimental data demonstrated detrimental effects of Cd stress on growth inhibition in seedlings. The effect of isosteviol and gibberellic acid in mitigating the symptoms of Cd toxicity in varying degrees in the presence of different parameters caused: more chlorophyll *a* *+* *b*, less malondialdehyde content in leaves, and higher dehydrogenase activity of root (except for the treatment of gibberellic acid) in wheat. Meanwhile, the treatment of isosteviol regulated the activity of antioxidant enzymes in leaves under Cd stress, which effectively removed the excess reactive oxygen species and reduced the oxidative damage to plant cells. Over all, this investigation provides a deeper understanding of the physiological response mechanism of Cd stress alleviation by added exogenous isosteviol in wheat.

## 5. Materials and Methods

### 5.1. Test Materials

Wheat plants utilized in our experiments were the *Triticum aestivum* L. cv. Yannong 19, selected from the Yantai Academy of Agricultural Sciences. *Triticum aestivum* L. cv. Yannong 19, a winter crop of wheat, was widely grown in Shandong province of China, which was characterized by the high vegetative mass, strong tillering, deep yellow-green leaf, as well as an in-field life cycle of approximately 245 days. The variety had strong disease resistance and drought resistance, but the cold resistance was general.

Stevioside was bought through the Stevioside Sweetener Factory of Nankai University (Tianjin, China). Isosteviol was derived from stevioside by means of acidic hydrolysis, based on the previously illustrated methods [63]. The resultant isosteviol was characterized through nuclear magnetic resonance (NMR) spectroscopy, infrared spectroscopy, as well as determining the melting point (212 °C), so as to confirm its structure. Besides, isosteviol purity (99%) was confirmed through high-performance liquid chromatography (HPLC). All reagents used in this study were analytical grade.

### 5.2. Experimental Design

#### 5.2.1. Tests for Analyzing the Wheat Germination in the Presence of Cd Stress

Seeds were subject to surface sterilization using 30% (*v*/*v*) H_2_O_2_, treated with Whatman paper that was imbibed within CdCl_2_ solutions at various Cd^2+^ concentrations (1, 10, 100, and 1000 µM) in Petri dishes (15 cm in diameter) and then incubated at 20 °C. For determining the seed germination rate, 50 seeds were tested in each treatment as part of one sample. The group of wheat seeds was grown in deionized water and considered as control. Hence, there were 12 replicate Petri dishes for each treatment in a fully randomized design. Seeds were considered germinated when the radicle visibly protruded through the seed coat. These germinated wheats were counted every day. If the amount of germination for wheat seeds did not increase for 3 days, germination was then considered complete. On day 7 following sowing, the FG rate and GI, together with VI, were computed using the following formulae. Then, the seedling number required was collected under all treatments to measure the RL as well as SL, along with FRN.

We also calculated the growth indexes based on the following formulae [64]:

FG rate = Total germinating seed number/Total tested seed number × 100%;(1)

FG—final germination rate of the sample per group harvested;
  GI = ΣGt/Dt;(2)
GI—germination index;
Gt—germinated seed number on day ‘t’;
Dt—corresponding germination day number;
t—germination time;
  VI = GI × S;(3)
VI—vigour index;
GI—germination index;
S—single plant length (cm).

#### 5.2.2. Tests for Determining the Effect of Isosteviol in Wheat Seed Germination in the Presence of Cd Stress

Based on the results gained from the above tests, germination of wheat seeds was inhibited when Cd^2+^ concentration reached 10 µM. Therefore, to study isosteviol effects on wheat seed germination in the presence of Cd stress, wheat seeds were processed at the 10 µM Cd^2+^ concentration. Subsequently, wheat seeds with the same size were picked out for tests; meanwhile, the seeds were subject to surface sterilization using 30% (*v*/*v*) H_2_O_2_, rinsed three times with deionized water, and then moisture was removed. Wheat seeds were divided into six batches, and all wheat seeds were added with four kinds of different concentrations of isosteviol (C_20_H_30_O_3_) (10^−10^, 10^−9^, 10^−8^ and 10^−7^ M) and GA (C_19_H_22_O_6_) (10^−5^ M), respectively. The group of wheat seeds was grown in only 10 µM Cd^2+^ solution and considered as Cd alone. The wheat seeds would later be imbibed for 24 h whilst keeping them away from light. Then, water was drained. Next, 50 seeds were added in each dish, followed by the placement of moistened filter paper at two layers into the Petri culture dishes. In these dishes, a 30 mL solution of 10 µM concentration containing Cd^2+^ was added. Twelve replicate Petri dishes for each treatment in a fully randomized design were obtained, followed by incubation at 20 °C. After 7 days of treatment, morphological parameters like FG rate, GI, VI, RL, SL, and FRN of wheat seedling were recorded. Plant tissues were randomly harvested, frozen in liquid nitrogen, and stored at −80 °C for biochemical analyses. For detection of chlorophyll, MDA, and antioxidant enzyme activity, freshly collected leaves were used. In addition, the dehydrogenase activity of root was also determined.

### 5.3. Sampling and Sample Analysis

#### 5.3.1. Determination of Chlorophyll

Chlorophyll was isolated from the fresh leaves using acetone (80%, *v*/*v*) and was determined according to Lichtenthaler (1987) [65]. Chlorophyll was extracted with 0.5 g small pieces (2 mm × 5 mm) of leaf tissue in 20 mL 80% acetone solution. After extraction at room temperature and dark conditions for 24 h, chlorophyll content in the supernatant was analyzed spectrophotometrically at 645 and 663 nm. (WFZ UV-2000, Unico^TM^ Shanghai Instrumentation Co., Shanghai, China).

Chlorophyll *a* (mg g^−1^) = [(12.7A_663_ − 2.69A_645_) × V]/(1000 × W); (4)

Chlorophyll *b* (mg g^−1^) = [(22.9A_645_ − 4.68A_663_) × V]/(1000 × W); (5)

Chlorophyll *a* + *b* (mg g^−1^) = [(20.2A_645_ + 8.02A_663_) × V]/(1000 × W); (6)

A—absorbance at different wavelengths;
W—fresh weight (g);
V—volume of extract solution (mL).

#### 5.3.2. MDA Contents

The concentration of MDA, which represents membrane LPO, was determined according to Heath and Packer’s method [66]. For this purpose, 0.5 g of fresh leaves were homogenized into 10% (*w*/*v*) trichloroacetic acid (TCA, 2 mL), followed by 15 min of centrifugation at 12,000× *g*. Subsequently, the formed reaction mixture was subject to 30 min of incubation in a 95 °C water bath, and 2 min of rapid cooling in the ice bath, followed by 10 min of centrifugation at 15,000× *g*. At this stage, supernatant absorbance was determined at the following wavelengths (including 532, 600, as well as 450 nm) by the spectrophotometer. MDA concentration was computed according to the equation below:

C = 6.45(A_532_ − A_600_) − 0.56A_450_; (7)

C—MDA concentration in µM;
A—absorbance at different wavelengths.

#### 5.3.3. Dehydrogenase Activity of Root

Dehydrogenase activity of root was measured using triphenyl tetrazolium chloride (TTC) assay [67]. In this method, young white roots were dried using the tissue paper before measuring the fresh mass. In total, 0.5 g of roots was put into the tubes containing 0.4% TTC (5 mL) as well as phosphate buffer (5 mL, 0.06 M, pH 7.0). Afterwards, the tubes were subject to 3 h of incubation at 37 °C, and a chemical reaction was developed and terminated through the addition of 1 M sulphuric acid (2 mL) into the tubes, following extraction of triphenyl formazan. In this regard, roots were transferred to the pestle containing ethylacetate (3‒4 mL) along with quartz sand, before grinding. Afterwards, the resultant liquid phase was transferred to the test tube. Then, ethylacetate was added until reaching the 10 mL mark, and absorbance at 485 nm was read by the UV-Vis recording spectrophotometer, which was then utilized in calculating the triphenyl tetrazolium formazan (TTF) levels. Moreover, dehydrogenase activity of every fresh root mass was determined according to the following method:

Dehydrogenase activity of root (μgTTF g^−1^ h^−1^ FW) = reduction in TTF. (8)

#### 5.3.4. Antioxidant Enzyme Activity

SOD activity was measured using nitroblue tetrazolium (NBT) assay [68]. To determine SOD activity, 3.0 mL reaction mixture was used containing 50 mM phosphate buffer (pH 7.8), 130 mM methionine, 750 µM NBT, 100 µM EDTA-Na_2_, 20 µM riboflavin, 50 µL enzyme extract, and 0.25 mL deionized water. Then the absorbance of the reaction mixture was measured with a spectrophotometer at 560 nm. One SOD unit was defined as the amount of enzyme required to inhibit the photoreduction of NBT by 50% [69], which was expressed in U g^−1^ fresh sample.

POD activity was measured using guaiacol oxidation assay [70]. To determine POD activity, 4 mL of the reaction mixture was used containing 100 mM phosphate buffer (pH 6.0), 5 mM guaiacol, 12.4 mM H_2_O_2_, and 1 mL enzyme extract. Then the absorbance of the reaction mixture was measured at 470 nm for 40 s. One POD unit was defined as 1 g fresh sample, leading to an absorbance (OD_470 nm_) value increase of 0.01 per minute [71], which was expressed in U min^−1^ g^−1^ fresh sample.

CAT activity was assayed following the method of Fathi et al. (2014) [72]. To determine CAT activity, 3 mL reaction mixture was used containing 150 mM phosphate buffer (pH 7.0), 50 mM H_2_O_2_, and 0.1 mL enzyme extract. Then the absorbance of the reaction mixture was measured at 240 nm for 40 s. One CAT unit was defined as 1 g of fresh sample, leading to an absorbance (OD_240 nm_) value decrease of 0.1 per minute [73], which was expressed in U min^−1^ g^−1^ fresh sample.

### 5.4. Statistical Analysis

Treatment effects were evaluated through variance analysis, employing the use of SAS statistical software package (version 9, SAS Institute, Cary, NC, USA). The differences in means were tested by the Fisher’s protected least significant difference (LSD), and the significance level was set at *p* < 0.05.

## Figures and Tables

**Table 1:** Effects of Cd on seed germination and seedling growth of wheat (*Triticum aestivum* L. cv. Yannong 19).

Cd^2+^ Concentration (µM)	FG Rate (%)	GI	VI	SL (cm)	RL (cm)	FRN
Control	96.3 ± 1.3 a	44.4 ± 0.1 a	528.4 ± 2.9 a	5.5 ± 1.0 a	6.4 ± 1.0 a	5.2 ± 0.7 a
1	96.2 ± 1.1 a	44.1 ± 0.6 a	516.0 ± 1.1 b	5.2 ± 0.9 ab	6.5 ± 1.2 a	5.0 ± 0.6 ab
10	91.3 ± 5.3 b	35.3 ± 0.4 b	388.3 ± 5.6 c	5.0 ± 1.1 b	6.0 ± 1.3 b	4.9 ± 0.7 b
100	86.0 ± 2.7 c	35.5 ± 0.2 b	266.3 ± 1.1 d	4.3 ± 0.6 c	3.2 ± 0.9 c	4.7 ± 0.9 c
1000	82.7 ± 0.9 d	33.3 ± 0.9 c	23.3 ± 2.0 e	0.5 ± 0.1 d	0.2 ± 0.1 d	1.3 ± 0.1 d

Cd: cadmium; FG: final germination; GI: germination index; VI: vigor index; SL: shoot length; RL: root length; FRN: fibrous root number; µM: Cd^2+^ concentration unit (µmol L^−1^). Data are presented in the form of mean ± S.D. (*n* = 12 in each group). Mean values with a same letter presented in the column represent the absence of significant difference (*p* < 0.05) upon Fisher’s least significant difference test.

**Table 2:** Effects of isosteviol on seed germination and seedling growth of wheat (*Triticum aestivum* L. cv. Yannong 19) under Cd stress.

Treatment (M)	FG Rate (%)	GI	VI	SL (cm)	RL (cm)	FRN
Control	96.1 ± 1.7 a	43.6 ± 0.4 a	562.4 ± 3.6 a	5.4 ± 1.1 b	7.5 ± 1.2 a	5.3 ± 0.6 a
Cd	91.7 ± 3.2 c	37.2 ± 0.7 d	431.5 ± 6.5 g	4.9 ± 0.3 cd	6.7 ± 0.4 c	4.3 ± 0.3 d
Cd + GA (10^−5^)	95.3 ± 2.8 ab	41.5 ± 0.9 b	522.9 ± 4.2 b	5.8 ± 1.0 a	6.8 ± 0.6 c	4.4 ± 0.2 cd
Cd + Iso (10^−10^)	92.0 ± 1.6 c	37.6 ± 0.6 d	451.2 ± 2.7 f	5.1 ± 0.8 c	6.9 ± 1.2 bc	4.5 ± 0.4 c
Cd + Iso (10^−9^)	93.8 ± 4.0 b	39.6 ± 0.2 c	491.2 ± 3.4 d	4.9 ± 0.6 cd	7.5 ± 1.1 a	4.6 ± 0.7 bc
Cd + Iso (10^−8^)	94.3 ± 2.2 b	39.5 ± 0.3 c	513.5 ± 5.5 c	5.4 ± 0.5 b	7.6 ± 0.7 a	4.7 ± 0.6 b
Cd + Iso (10^−7^)	93.3 ± 3.8 bc	38.5 ± 0.5 cd	465.9 ± 4.7 e	5.1 ± 0.9 c	7.0 ± 0.5 b	4.4 ± 0.3 cd

Cd: cadmium; GA: gibberellic acid; Iso: isosteviol; FG: final germination; GI: germination index; VI: vigor index; SL: shoot length; RL: root length; FRN: fibrous root number; M: isosteviol and gibberellic acid concentration unit (mol L^−1^). Data are displayed in the form of mean ± S.D. (*n* = 12 in each group). Mean values with a same letter presented in the column represent the absence of significant difference (*p* < 0.05) upon Fisher’s least significant difference test.

**Table 3:** Effects of isosteviol on chlorophyll contents in wheat (*Triticum aestivum* L. cv. Yannong 19) leaves under Cd stress.

Treatment (M)	Chlorophyll (mg g^−1^)			Chlorophyll *a*/*b*
	Chlorophyll *a*	Chlorophyll *b*	Chlorophyll *a* + *b*	
Control	1.7 ± 0.3 a	0.7 ± 0.1 a	2.4 ± 0.2 a	2.4 ± 0.2 ab
Cd	0.9 ± 0.2 d	0.4 ± 0.04 bc	1.3 ± 0.2 e	2.3 ± 0.2 ab
Cd + GA (10^−5^)	1.2 ± 0.1 b	0.5 ± 0.06 b	1.7 ± 0.1 b	2.4 ± 0.3 ab
Cd + Iso (10^−10^)	0.9 ± 0.1 d	0.4 ± 0.03 bc	1.3 ± 0.1 e	2.3 ± 0.2 ab
Cd + Iso (10^−9^)	1.0 ± 0.1 c	0.4 ± 0.04 bc	1.4 ± 0.1 d	2.5 ± 0.1 a
Cd + Iso (10^−8^)	1.1 ± 0.04 bc	0.5 ± 0.1 b	1.6 ± 0.2 bc	2.2 ± 0.1 ab
Cd + Iso (10^−7^)	1.1 ± 0.1 bc	0.4 ± 0.03 bc	1.5 ± 0.1 c	2.7 ± 0.4 a

Cd: cadmium; GA: gibberellin acid; Iso: isosteviol; M: isosteviol and gibberellic acid concentration unit (mol L^−1^). Data are presented in the form of mean ± S.D. (*n* = 12 in each group). Mean values with a same letter presented in the column represent the absence of significant difference (*p* < 0.05) upon Fisher’s least significant difference test.

## Data Availability

The data presented in this study are available within the article and its Appendix A.

## Abbreviations

cadmium: Cd; gibberellic acid: GA; malondialdehyde: MDA; superoxide dismutase: SOD; peroxidase: POD; catalase: CAT; reactive oxygen species: ROS; plant growth regulators: PGRs; abscisic acid: ABA; indole-3-acetic acid: IAA; gibberellins: GAs; salicylic acid: SA; isosteviol: Iso; final germination: FG; germination index: GI; vigor index: VI; root length: RL; shoot length: SL; fibrous root number: FRN; lipid peroxidation: LPO; trichloroacetic acid: TCA; triphenyl tetrazolium chloride: TTC; triphenyl tetrazolium formazan: TTF; nitroblue tetrazolium: NBT.

## Appendix A

**Table A1:** Effects of isosteviol on MDA content, root dehydrogenase activity, and antioxidant enzyme activity of wheat (*Triticum aestivum* L. cv. Yannong 19) seedlings under Cd stress.

Treatment (M)	MDA Concentration (nmol g^−1^ FW)	Dehydrogenase Activity of Root (μg TTF g^−1^ h^−1^ FW)	SOD Activity (U g^−1^ FW)	POD Activity (U min^−1^ g^−1^ FW)	CAT Activity (U min^−1^ g^−1^ FW)
Control	5.8 ± 0.9 d	178.6 ± 3.9 a	35.8 ± 2.4 e	130.5 ± 4.6 f	23.1 ± 0.9 a
Cd	18.9 ± 0.7 a	140.3 ± 3.7 d	40.6 ± 2.8 d	150.5 ± 7.8 e	14.6 ± 2.1 de
Cd + GA (10^−5^)	15.7 ± 1.2 c	142.6 ± 4.2 d	54.3 ± 3.4 b	195.8 ± 6.4 b	23.6 ± 1.0 a
Cd + Iso (10^−10^)	18.0 ± 1.1 a	156.3 ± 4.1 c	45.2 ± 3.9 c	180.5 ± 5.9 d	15.2 ± 1.0 d
Cd + Iso (10^−9^)	16.4 ± 0.7 b	165.5 ± 2.7 b	47.6 ± 3.5 c	178.6 ± 9.5 d	22.2 ± 1.5 ab
Cd + Iso (10^−8^)	15.6 ± 1.0 c	160.3 ± 6.0 bc	58.6 ± 2.6 a	210.3 ± 4.6 a	21.9 ± 1.2 b
Cd + Iso (10^−7^)	16.6 ± 1.1 b	158.7 ± 2.1 c	57.6 ± 3.2 a	189.6 ± 7.2 c	17.6 ± 0.9 c

MDA: malondialdehyde; Cd: cadmium; GA: gibberellic acid; Iso: isosteviol; M: isosteviol and gibberellic acid concentration unit (mol L^−1^); SOD: superoxide dismutase; POD: peroxidase; CAT: catalase; FW: fresh weight. Data are displayed in the form of mean ± S.D. (*n* = 12 in each group). Mean values with the same letter presented in the column represent the absence of significant difference (*p* < 0.05) upon Fisher’s least significant difference test.
